# Comparative Study on the Corrosion Resistance of 6061Al and SiC_3D_/6061Al Composite in a Chloride Environment

**DOI:** 10.3390/ma14247730

**Published:** 2021-12-14

**Authors:** Liang Yu, Shuangshuang Hao, Xiaodong Nong, Xiuling Cao, Chen Zhang, Yuan Liu, Yulong Yan, Yanli Jiang

**Affiliations:** 1 Key Laboratory of New Processing Technology for Nonferrous Metals & Materials, Guilin University of Technology, Guilin 541004, China; 2010054@glut.edu.cn (L.Y.); sshao98@163.com (S.H.); zc405716298@163.com (C.Z.); 17853310445@163.com (Y.L.); yyl_211200313@163.com (Y.Y.); 2 Guangxi Scientific Experiment Center of Mining, Metallurgy and Environment, Guilin 541004, China; 3 Collaborative Innovation Center for Exploration of Nonferrous Metal Deposits and Efficient Utilization of Resources, Guilin University of Technology, Guilin 541004, China; 4 Jihua Laboratory, Foshan 528200, China; nxdpg8@126.com; 5 Hebei Technology Innovation Center for Intelligent Development and Control of Underground Built Environment, School of Exploration Technology and Engineering, Hebei Geosciences University, Shijiazhuang 050031, China

**Keywords:** SiC_3D_/6061Al composite, corrosion, Cl^−^, electrochemistry, salt spray test

## Abstract

Interface problems and the destruction of the continuity of the oxide film in the Al matrix usually reduce the corrosion resistance of the material. In this paper, the corrosion resistance of Al matrix composites (AMCs) was improved by introducing the silicon carbide skeletons (SiC_3D_) obtained with polymer replica technology. SiC_3D_/6061Al was fabricated by infiltrating molten 6061Al alloy in the oxidized SiC_3D_ using the low-pressure casting method. The corrosion resistance performances of 6061Al and SiC_3D_/6061Al in NaCl solution were studied by electrochemical, neutral salt spray corrosion (NSS), and salt leaching (SL) tests. Results show corrosion resistance of SiC_3D_/6061Al is higher than that of 6061Al alloys by open circuit potential (OCP), potentio-dynamic polarization (PDP), and electrochemical impedance spectroscopy (EIS) tests. However, NSS and SL tests show the corrosion resistance of SiC_3D_/6061Al is lower than that of 6061Al alloy. The reason is a corrosion resistant and anti-oxidation network macrostructure with large interface recombination, few concentrated interfaces, and a small specific area that formed in SiC_3D_/6061Al. SiC_3D_ cannot damage the continuity of the Al_2_O_3_ passivating film, and the network macrostructure greatly improves the corrosion resistance performance.

## 1. Introduction

Ceramic-reinforced metal matrix composites (MMCs) mainly include ceramic particle-reinforced MMCs and ceramic skeleton-reinforced MMCs [1,2]. In recent years, research on the corrosion behavior of silicon carbide (SiC) particles-reinforced aluminum matrix composites (AMCs) has received extensive attention. However, SiC particles are difficult to disperse uniformly in the AMCs due to the limitations of the preparation technology [3,4]. The inhomogeneity of the SiC particles’ dispersion may lead to weak parts in the material that severely reduce the corrosion resistance. In addition, high residual stresses are generated at the composite interface during the preparation process due to the mismatch of the expansion coefficient and other physical properties between the Al alloy matrix and the SiC particles. An increase in residual stress causes significant changes in the microstructure and properties of the Al alloys near the interface, which significantly reduces the corrosion resistance of the material [5,6,7,8].

To improve the corrosion resistance of the AMCs, SiC particles were made into three-dimensional reticulated porosity SiC skeletons (SiC_3D_), and compounded with Al alloys to produce a SiC_3D_/Al alloy composite [7,8,9]. The stiffness of SiC_3D_/Al alloy composite is superior to that of Al alloy material, and its toughness and structural integrity are superior to those of monolithic SiC ceramic materials. SiC_3D_/Al alloy composites are defined as multiphase composites in which SiC_3D_ and Al alloy are topologically interconnected. SiC_3D_/Al alloy composite is also called interpenetrating phase composite (IPC) or co-continuous phase composite [10,11,12]. The interface of SiC_3D_/Al alloy has low energy, which inhibits the nucleation and growth of columnar crystals and does not produce segregation.

The 6061Al alloy is a precipitation hardening alloy with a high content of Mg and Si, widely used in engineering applications including transport and construction. Its superior corrosion resistance makes it a suitable candidate material for marine structural applications. The increasing demand for light-weight and excellent mechanical properties (such as integration, high wear resistance, high tensile strength, high hardness, and high corrosion resistance) of materials has led to the fabrication of SiC_3D_/6061Al composites. Compared with particle-reinforced SiC_P_/Al composites, SiC_3D_/6061Al have fewer defects (such as porosity and shrinkage cavity) in the casting process. The contact interface area of SiC_3D_ and Al alloy in SiC_3D_/6061Al composites is larger, the interface number is less, and more concentrated than those of SiC_P_/Al composites. These characteristics improve the corrosion resistance of SiC_3D_/6061Al composites.

We fabricated SiC_3D_/6061Al composite by infiltrating molten 6061Al alloy to fill the oxidized SiC_3D_ ung a low-pressure casting process. The corrosion resistance performances of 6061Al and SiC_3D_/6061Al composite in NaCl solution were studied using electrochemical and salt spray tests in simulated neutral ocean environments in this paper.

## 2. Materials and Methods

### 2.1. Experimental Materials

SiC_3D_ skeletons were fabricated by the polymer replica method [10,11,12,13]. A polyurethane open-cell sponge template (Shenzhen Lvchuang Environmental Filter Material Co., Ltd., Shenzhen, China) with 10 PPI (10 pores/inch) and dimensions of 360 × 280 × 7.5 mm^3^ was immersed into the as-prepared SiC slurry, followed by passing through a preset roller (XL-KLYP4, Dongguan Xilong Electrical machinery Equipment Co., LTD, Dongguan, China) to remove the excess slurry. A-SiC powder (d_5o_ < 0.3 μm, purity > 98%, *p* = 3.18 g/cm^3^, from Shandong Jinmeng New Material Co., Ltd., Yantai, China) was used for preparing SiC coating slurry. The remaining closed pores were eliminated by blowing compressed air through the sponge structure. The sponge was microwave dried for 15 min to produce green SiC reticulated porosity bodies with good handling strength, sintered at 2050 °C for 0.5 h in a graphite resistance furnace (Jinzhou Santai Electric Furnace Factory, Jinzhou, China), with argon gas as the sintering atmosphere to prepare the SiC_3D_ skeletons. The SiC_3D_ skeletons were heated at 1200 °C for 4 h in stationary ambient air (Shanghai Jvjing Precision Instrument Manufacturing Co., Ltd., Shanghai, China). The SiC_3D_/Al composite was prepared via casting the liquid Al alloy (Yunnan Aluminum Co., Ltd., Kunming, China) was cast into the oxidized SiC_3D_ skeletons at 750 °C by the low-pressure casting equipment (Zhejiang Wanfeng Technology Development Co., Ltd., Xinchang, China) [14]. Table 1 shows the chemical composition of the 6061Al alloy. The T6 heat treatment for SiC_3D_/6061Al included solution treatment quench and artificial aging using a heat treating furnace (Shanghai Jvjing Precision Instrument Manufacturing Co., ltd., Shanghai, China). The preparation process of the composites was described in detail in [13,14]. The specimens were machined from SiC_3D_/6061Al and compared with 6061Al alloy to study their corrosion performance.

### 2.2. Characterization

The potentio-dynamic polarization (PDP) test and electrochemical alternating current impedance spectroscopy (EIS) were measured using an electrochemical workstation (CHI 760E, Shanghai Chenhua Instrument Co., LTD, Shanghai, China) with a three-electrode system: a standard calomel electrode (SCE) as the reference electrode, a platinum plate as the auxiliary electrode, and the sample as the working electrode. The SiC_3D_/6061Al and 6061Al alloy specimens were fabricated to a 10 × 10 × 2 mm^3^ dimension. A copper wire (Jiangsu Jinzixuan Metal Technology Co., LTD, Wuxi, China) was connected to the back of each sample prior to embedding in an epoxy resin (Guangzhou Weiyi Metallographic Test Instrument Co., LTD, Guangzhou, China). The working area exposed to the solution was 1 cm^2^. The specimen surfaces were abraded on 1500 SiC paper and washed with distilled water [15,16,17,18]. Open circuit potential (OCP) was measured with polished samples for 6 min. Experiments were performed at room temperature in a glass cell containing 3.5 wt% NaCl solution (Dongguan xunye Chemical Reagent Co., LTD, Dongguan, China). PDP experiments were carried out after the open circuit potential remains stable by sweeping the potential from the cathodic to the anodic direction at a scan rate of 0.25 mV/s, with an interval of about ±300 mV relative to the self-corrosion potential. EIS was measured using a 10 mV perturbation potential sine wave under OCP with a frequency range of 10^−2^–10^5^ Hz for the uncorroded sample. EIS was measured with a frequency range of 10^−3^–10^4^ Hz for the corroded samples at different salt spray corrosion times. The test data were fitted by ZView software (Version3.1, 2007, Scribner Associates Inc., Southern Pines, NC, USA) [17,18,19,20].

Neutral salt spray corrosion (NSS) tests were measured using an automatic salt spray testing machine (ZK-60K, Dongguan Zhenke Testing Equipment Co., LTD, Dongguan, China) with a neutral NaCl solution of 5 wt% corrosive liquid and pH 6.5–7.2. The experiment periods were 24, 72, 144, 168, and 240 h at (35 ± 1) °C.

All the specimens were cold mounted and polished with polishing papers (Zibo Bingyang Grinding Technology Co., LTD, Zibo, China). The samples were further mirror-polished using diamond pastes. (Zibo Bingyang Grinding Technology Co., LTD, Zibo, China). The specimens were polished with coarse diamond pastes (W20), (W14), (W7) and (W3.5), and then polished with fine diamond gypsum (W1) to achieve mirror-polished surface. The specimens were etched with Keller’s solution of 95 mL deionized water + 2.5 mL HNO_3_ + 1.5 mL HCl + 1.0 mL HF prepared in our laboratory. Etched the sample with Keller’s solution for 10-20 s and rinsed with warm water. It can then enter concentrated HCl to enhance the profile of all components. Microstructural evaluations of samples and the corrosion products were characterized using a Zeiss GeminiSEM 300 field emission scanning electron microscope (FESEM, Oberkochen, Germany) equipped with an energy dispersive X-ray spectrometer (EDS, Oberkochen, Germany).

The salt leaching (SL) tests simulated the marine environment, using 3.5 wt %, pH 6.6 neutral NaCl solution for immersion, the test period was 21, 61, and 150 days, the test temperature was (25 ± 2) °C, and after immersion corrosion, the method of removing corrosion products was the same as NSS and used Zeiss GeminiSEM 300 to determine the corrosion products and observe the corrosion morphology.

The density and porosity of the materials were determined by Archimedes’ method.

## 3. Results

### 3.1. Uncorroded Microstructure of the Two Materials

**Figure 1:**
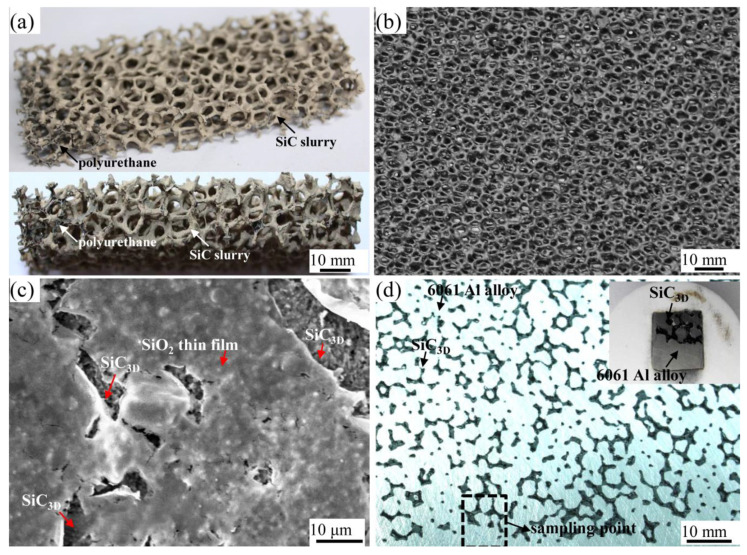
(**a**) Green SiC_3D_ skeletons; (**b**) sintered SiC_3D_ skeletons; (**c**) SiO_2_ thin film was formed on the SiC_3D_ skeletons; (**d**) sampling points of SiC_3D_/6061Al.

Figure 1a shows green SiC_3D_ skeletons, in which the black network material is polyurethane sponge, and the coated yellow hard shell is dried SiC slurry. SiC particles were sintered to be a rigid ceramic network skeleton shown in Figure 1b. The pores of SiC_3D_ skeletons are approximate hexagonal holes, interconnected, and 2–3 mm in size. A SiO_2_ thin film was formed on the SiC_3D_ struts shown in Figure 1c. Figure 1d shows the sample used in the experiment from the SiC_3D_/6061Al. The brighter phase is the Al alloy matrix, and the darker is the SiC_3D_ skeletons’ strengthening phase. The T6 heat-treated SiC_3D_/6061Al macrostructure shows the molten Al alloy infiltrates the SiC_3D_ skeletons completely. The density measurement indicates that SiC_3D_/6061Al contains about 35 vol.% SiC and about 65 vol.% Al alloy, and the density of SiC_3D_/6061Al is 99.1% of the theoretical density. SiC_3D_/6061Al exhibits uniform and interconnected structures. The appearance of the phases suggests that the microstructures are in agreement with IPCs SiC/Al described in [1,5,6,7,8,9]. The continuous 6061Al alloy and the SiC_3D_ skeletons are topologically interconnected in the three-dimensional space, forming an anti-corrosion, anti-oxidation network [13].

**Figure 2:**
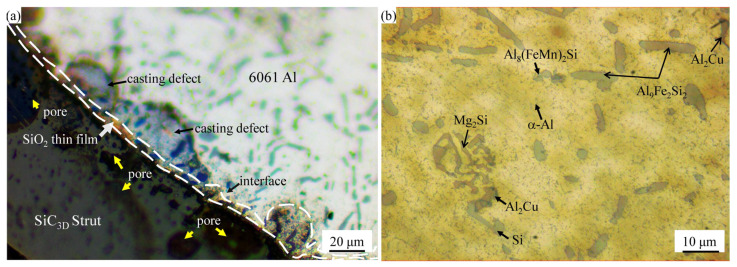
Optical micrograph (OM) image of two materials: (**a**) SiC_3D_/6061Al; (**b**) 6061Al alloy.

Figure 2a shows a representative optical micrograph (OM) of the T6 heat treatment SiC_3D_/6061Al. The thickness of 3–5 μm SiO_2_ layers are found at the interface of the infiltrated SiC_3D_ skeletons’ strengthening phase and Al alloy in Figure 2a, which could effectively prevent direct contact between them and form Al_4_C_3_. We did not observe Al_4_C_3_ in SiC_3D_/6061Al composites, indicating that SiC_3D_ cannot decompose to Si and C in a high-temperature environment during low-pressure casting. The surface oxidization of SiC_3D_ can suppress the interfacial reaction and improve the interface bonding strength of SiC_3D_/6061Al composites. Eutectic Si in a basic hypoeutectic Al–Si microstructure is observed in the Al alloy matrix of SiC_3D_/6061Al. Secondary dendrite arm spacing (SDAS) with a maximum size of 20 μm was obtained by low-pressure casting and T6 heat treatment. The presence of rod-like and spherical precipitates (second phase particles) was observed in the grain boundaries and interdendritic region of α-Al. The organization at the interface is disordered and has a high entropy value, so it is more susceptible to corrosion. Due to their nano size, the strengthened intermetallic precipitates induced by T6 heat treatment cannot be detected by OM.

Figure 2b shows the OM of 6061Al alloy. Compared with the microstructure of SiC_3D_/6061Al, the grains are larger, and the number of second phase particles is smaller. In the phase composition of the α-Al-matrix, the rod-like and spherical Si, Al_8_(FeMn)_2_Si, Al_9_Fe_2_Si_2_, Al_2_Cu, and “Chinese characters” shaped Mg_2_Si, formed during solidification remained undissolved during the T6 heat treatment [13]. Al_8_(FeMn)_2_Si and Al_9_Fe_2_Si_2_ generally small size (less than 10 μm), appeared lighter in color in comparison with Mg_2_Si and Al_2_Cu phases. SDAS with a maximum size of about 25 μm is obtained by the low-pressure casting method and T6 heat treatment.

### 3.2. Polarization Curve

**Figure 3:**
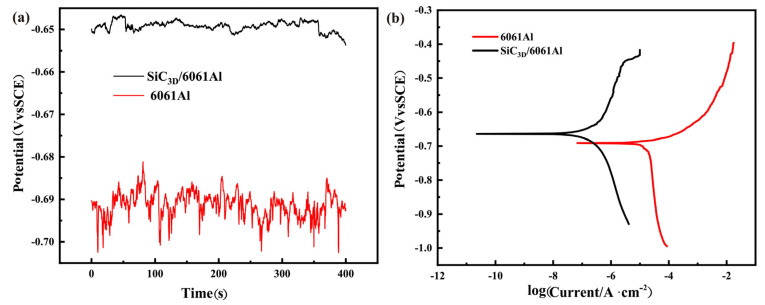
(**a**) Open circuit potential (OCP) curve; (**b**) potentio-dynamic polarization (PDP) curve.

The electrochemical corrosion of SiC_3D_/6061Al composite includes the metal phase and interface corrosion, and the ceramic phase does not participate in corrosion. Figure 3a describes the OCP and PDP of SiC_3D_/6061Al and 6061Al in NaCl solution. Table 2 shows the OCP, corrosion potential (*E*_corr_), and corrosion current density (*I*_corr_) values obtained from the curve in Figure 3. The OCP fluctuation of SiC_3D_/6061Al composite is smaller than that of 6061Al alloy. In the polarization curve, the SiC_3D_/6061Al composite shows a larger *E*_corr_ value and a smaller *I*_corr_ value than 6061Al, revealing that SiC_3D_/6061Al composite has better corrosion resistance than 6061Al.

**Figure 4:**
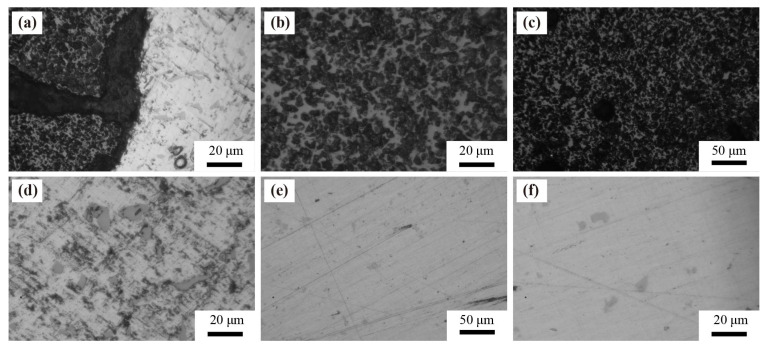
Optical micrographs of two materials after Tafel corrosion: (**a**–**d**) SiC_3D_/6061Al; (**e**,**f**) 6061Al alloy.

Figure 4 shows the optical micrograph after electrochemical corrosion. More Mg_2_Si particles were precipitated in the SiC_3D_/6061Al composite (Figure 4d) than 6061Al alloy (Figure 4f). Cu formed an intermetallic phase with Al that precipitates during solidification either as blocky Al_2_Cu or as alternating lamellae of α-Al + Al_2_Cu. Mg was present as Mg_2_Si in 6061Al alloy for Mg was not in the solution. The corrosion tendency and the sensitivity of early pitting micro-pore nucleation of SiC_3D_/6061Al is low due to the small potential difference between Mg_2_Si particles and the 6061Al matrix. Mg_2_Si particles block the continuity of the matrix in the middle and later stages of corrosion, inhibiting further corrosion [18,19,20,21,22]. Moreover, the second phase precipitation reduces the pitting nucleation sensitivity of the SiC_3D_/6061Al, inhibits the corrosion tendency, and increases the corrosion potential. It is worth noting that an anti-corrosion, anti-oxidation network macrostructure with advantages of large interface recombination, few concentrated interfaces, and the small specific area is formed in SiC_3D_/6061Al. Thus, the corrosion resistance of SiC_3D_/6061Al is higher than that of 6061Al alloy.

### 3.3. Electrochemical Alternating Current Impedance Spectroscopy (EIS) of Uncorroded Materials

**Figure 5:**
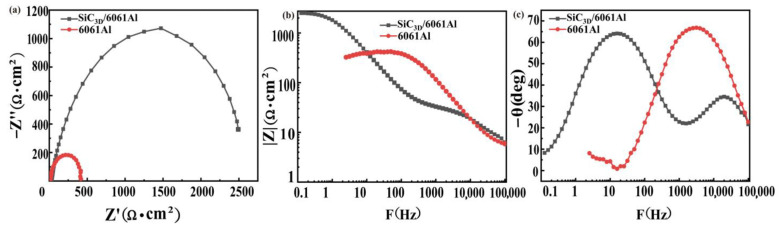
Uncorroded electrochemical alternating current impedance spectroscopy (EIS): (**a**) Nyquist diagram; (**b**) Bode diagram (|Z|-F); (**c**) Bode diagram (−θ-F).

Figure 5 shows the EIS of SiC_3D_/6061Al and 6061Al without salt spray corrosion. The Nyquist diagrams of the two materials are both capacitive reactance diagrams (Figure 5a). The impedance spectrum is shown as a capacitive reactance arc in the high-frequency region, reflecting the corrosion electrochemical reaction on the electrode surface. The actual shrinking tail appears in the low-frequency area, which the adsorption of the reaction may cause intermediate products at the electrode surface [23,24]. The Nyquist diagram in Figure 5a is a semicircle; thus, the control steps of the electrode process are determined by the electrochemical reaction step (charge transfer process). The impedance caused by the diffusion process can be ignored. Figure 5b,c show the Bode diagram has two time constants. Compared to 6061Al, the impedance modulus of SiC_3D_/6061Al is higher in the early stage of corrosion, lower in the middle stage, and slightly higher in the later stage. The phase angle of SiC_3D_/6061Al is higher than that of the 6061Al alloy in the early stage. However, it is lower than that of the 6061Al alloy in the middle and late stages. EIS results of the SiC_3D_/6061Al and 6061Al are consistent with the PDP results in Figure 3.

### 3.4. Corrosion Morphology Analysis

**Figure 6:**
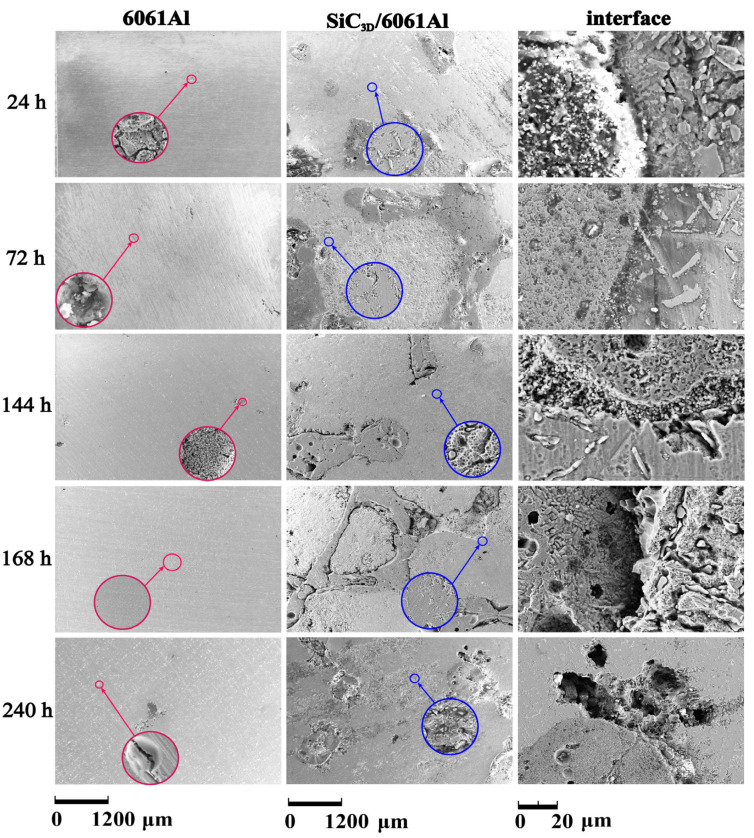
Corrosion morphology of SiC_3D_/6061Al and 6061Al under different times after NSS (neutral salt spray) corrosion.

The corrosion degree of SiC_3D_/6061Al and 6061Al alloy gradually reduces at extended times, as shown by periodic NSS tests. The corrosion process combines pitting corrosion and intergranular corrosion and eventually develops into exfoliation corrosion [23,24,25]. Figure 6 shows the surface morphology of the SiC_3D_/6061Al and 6061Al samples corroded in NSS tests. In order to observe the fine structure of corrosion degree of the 6061Al alloy and the metallic phase of SiC_3D_/6061Al composite, the metal phases of the two materials were zoomed.

After 24 h, the corrosion of 6061Al alloy is the most severe, with many pitting corrosion pits after 24 h.

After 72 h, pitting corrosion pits merged, forming a ravine shape, the pitting area increased, and some areas peeled off due to the accumulation of pitting corrosion pits. The irregular and uneven distribution of grains in the internal area of 6061Al alloy is discovered in the magnification of pitting corrosion pits, which is directly related to the vertical development rate of pitting corrosion greater than the horizontal development.

After 144 h, the development of horizontal corrosion merged multiple originally developed independent pitting corrosion pits, making the inner surface of pitting pits irregular. With increasing corrosion time, large areas of pitting corrosion occurred with an irregular round or elliptical outline and was discovered in 6061Al alloy. In the SiC_3D_/6061Al composite, the large potential difference between Al alloy and SiC_3D_ provides a driving force for pitting corrosion leading to crevice formation. The interface of SiC_3D_/6061Al is corroded and reduced.

After 168 h, 6061Al alloy changed from pitting to uniform corrosion, and deep cracks appeared at the interface of the SiC_3D_/6061Al composite.

After 240 h, large and deep corrosion pits existed in the metal phase at the SiC_3D_/6061Al composite interface, causing catastrophic corrosion. A large area of 6061Al alloy spalled, and large cracks appeared on the surface of the material.

Therefore, the corrosion tendency of the SiC_3D_/6061Al composite before and during the corrosion is less than that of the 6061Al alloy. However, the reinforcement clustering and high intermetallic volume fraction provide a large cathodic area in the composite, causing susceptibility to pit. In the later corrosion period, exfoliation corrosion occurs at the interface of the SiC_3D_/6061Al composite material, resulting in corrosion pits. Cl^−^ is immersed in the corrosion pits to further aggravate the corrosion.

### 3.5. Microstructure of Corrosion Products

**Figure 7:**
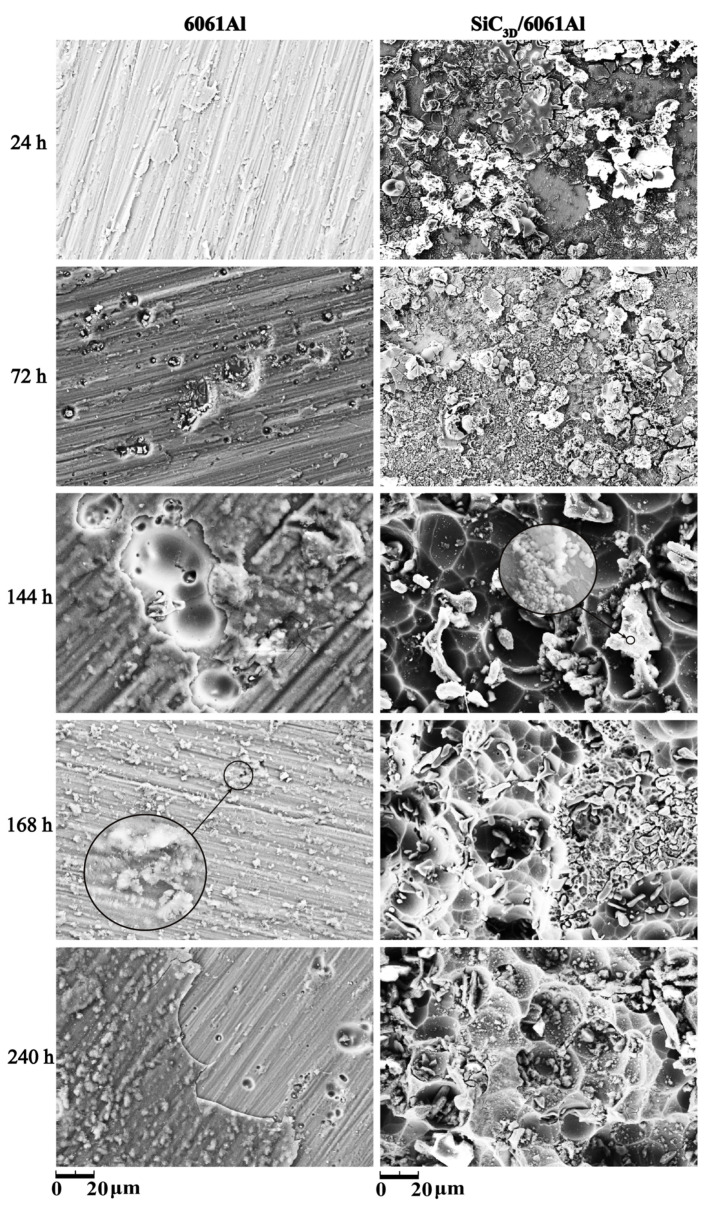
Corrosion product morphology under different times after NSS corrosion.

Figure 7 shows the corrosion product morphology of the SiC_3D_/6061Al and 6061Al samples corroded in NSS tests. Observation of the samples after NSS showed that the corrosion products of the 6061Al alloy accumulated on the surface of the samples in the form of a flaky salt spray corrosion for 24 h. The corrosion products gradually increased with increasing time. After 144 h, the corrosion products gradually increased as time passed. After 168 h, the 6061Al alloy undergoes a secondary reaction to form an Al_2_O_3_ film, which improves the passivating performance of the metal, thereby improving the corrosion resistance [26,27]. After 240 h, the passivation film was destroyed, and cracks appeared on the surface of the 6061Al.

**Figure 8:**
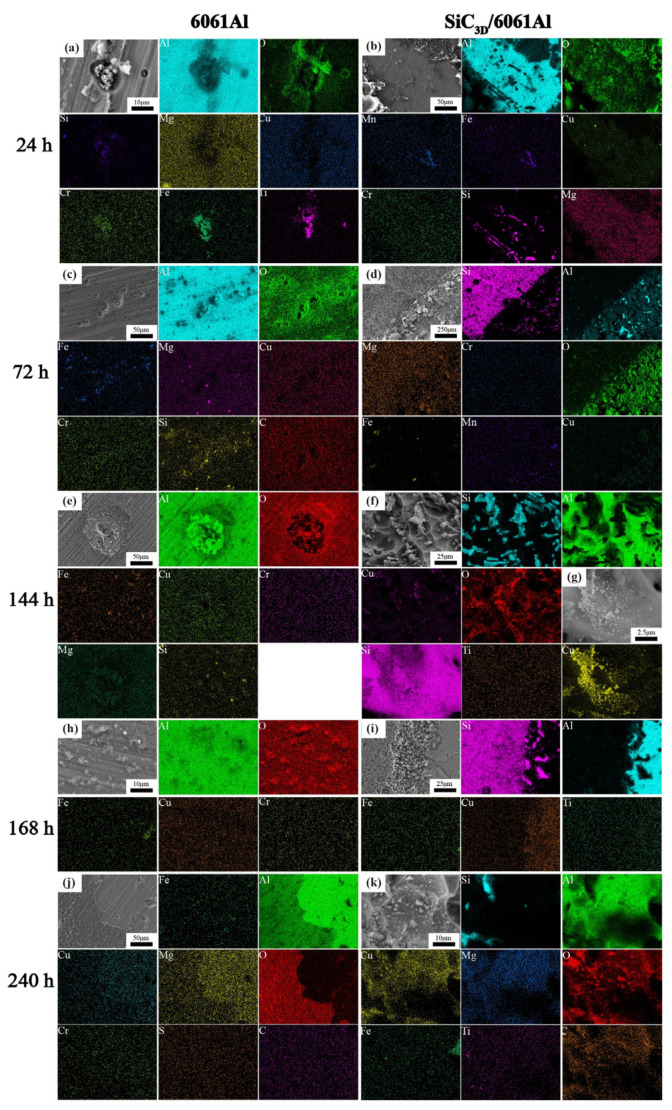
Energy dispersive X-ray spectrometer (EDS) of corrosion products with different times after NSS corrosion. (**a**,**c**,**e**,**h**,**j**) 6061Al; (**b**,**d**,**f**,**g**,**i**,**k**) SiC_3D_/6061Al.

Figure 8 shows the corrosion product EDS under different times after NSS corrosion. The scale bar used for the elements is the same as that used for the corresponding microstructure. After 24 h, a thin and dense corrosion product layer was formed on the surface of the SiC_3D_/6061Al composite. The corrosion product layer continues to thicken with increasing corrosion time. The corrosion products crack into irregular flakes, with the increasing thickness of the loose layer and the number of pores, and the bonding strength between the loose layer and the substrate decreased. Moreover, the electrochemical reaction in the pores was intensified. Under the agitation of the anode bubbles, the loose layer was separated from the substrate, and a large area of the substrate was exposed. There is a potential difference between the second phase particles precipitated along the grain boundary and the matrix, which constitutes a corrosion battery, causing the matrix around the second phase particles to dissolve and intergranular corrosion to occur. With the charge transfer, Fe and Cu continued to precipitate. The second phase particles dissolved by themselves, which expanded the corrosion pits.

After 144 h, the Cu element in the Al matrix increase was beneficial to increase the electrode potential and improve the corrosion resistance. Therefore, after 144 h, the degree of corrosion of the metal phase was not evident.

After 168 h, EDS of the surface of the 6061Al alloy in Figure 8j reveals that the Mg and Cu elements are contained in the bare substrate. The damaged Al_2_O_3_ film is in the active state as the anode, and the undamaged film remains in the passive state as the cathode, which constitutes an activation–passivation battery [26,27]. The oxidation-reduction reaction dissolves the metal in the pore to maintain the electrical neutrality in the pore, Cl^−^ migrates into the pores, and the pH value decreases. Under the action of H^+^ and Cl^−^, the metal is activated, forming a pore activation (inside)–passivation (outside) corrosion battery, and the migration of Cl^−^ increases. The pH drops further, promoting further corrosion. The product on the outer surface is Al(OH)_3_, which further decomposes into Al oxides, occasionally containing some chlorine, sulfur, and mineral salt elements, consistent with the surface EDS analysis.

The SiC_3D_/6061Al composite mainly exhibits pitting corrosion at the interface, while the 606lAl alloy with the same composition undergoes uniform corrosion. The SiC_3D_/6061Al corrodes faster than the 6061Al alloy. Further analysis shows that the thin layer of interface reaction products formed at the interface of the SiC_3D_/6061Al acts as a cathode and leads to local galvanic corrosion at the interface.

In the early stage of corrosion, the corrosion products protect the substrate, but they gradually weaken as the corrosion time increases. In the later stage, the electrode potential decreases, and the corrosion tendency increases for the loose layer is separated from the substrate, chloride directly contacts the exposed substrate, and the copper atom content at the interface decreases. NSS tests show that the boundary is the key part that causes high corrosion current density in the metal matrix composite material. The SiC particles at the boundary of the SiC_3D_/6061Al composite material can be used as the cathode site for oxygen reduction, increasing the area of oxygen reduction, resulting in an increase corrosion rate (CR). Catastrophic corrosion at the interface leads to an increase in the overall CR of SiC_3D_/6061Al composite. The reason for the higher CR of the composite may be the galvanic corrosion or crack corrosion between the aluminum matrix and the framework (interface silicon carbide) to cause the corrosion of aluminum.

The protective performance of surface corrosion products first strengthens, then weakens, and finally strengthens. The corrosion products in the early stage of corrosion protect the substrate. As the corrosion time increases, it shows a gradual weakening trend. In the later stage, the loose layer is separated from the substrate, and the large area of the substrate is exposed. Chloride ions are in direct contact with the substrate, and the content of copper atom at the interface is small. The electrode potential decreases and the corrosion tendency increases.

### 3.6. EIS of Corrosion Product

**Figure 9:**
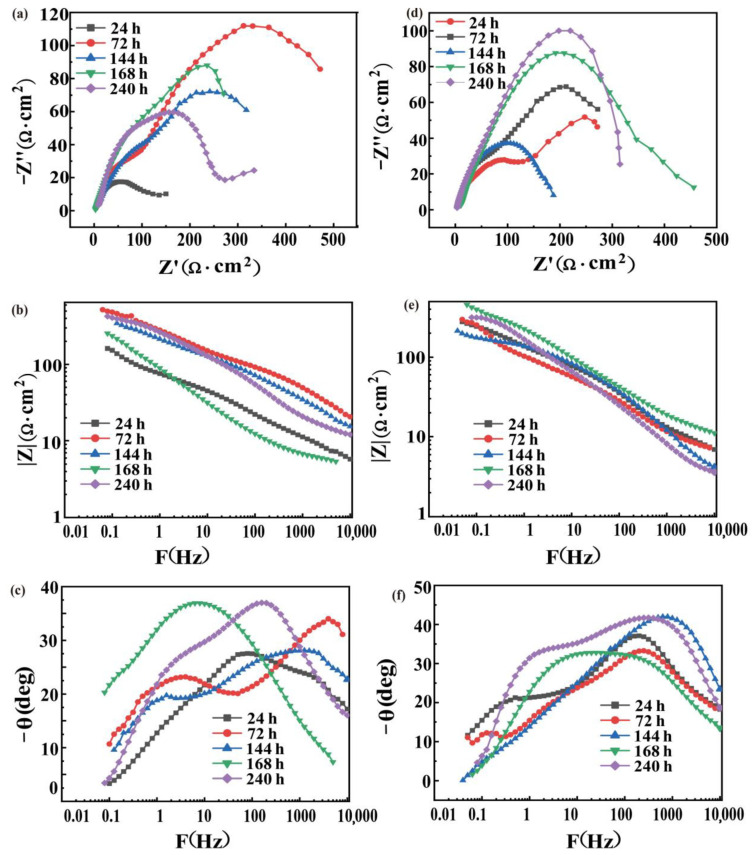
EIS of SiC_3D_/6061Al (**a**–**c**) and EIS of 6061Al alloy (**d**–**f**) after NSS at different times: (**a**,**d**) Nyquist diagram; (**b**,**e**) Bode diagram (|Z|-F); (**c**, **f**) Bode diagram (−θ-F).

To study the effect of the corrosion product layer on the surface of the material on the corrosion process, the EIS of the corrosion product is shown in Figure 9. The Nyquist diagram of SiC_3D_/6061Al is composed of two capacitive reactance diagrams (Figure 9a). The high and intermediate frequency capacitive resistance arc corresponds to the corrosion products on the surface of the SiC_3D_/6061Al. The low-frequency capacitive reactance arc corresponds to the electrochemical corrosion reaction of the electrode surface. The capacitive resistance arc in the high-frequency region slowly increased, and corrosion products slowly formed on the surface of SiC_3D_/6061Al with increasing salt spray time. The electrochemical reaction on the SiC_3D_/6061Al surface in the early stage of corrosion was violent, and the surface electrochemical reaction slowed down with the formation of corrosion products. Figure 9b,c show that the Bode diagrams have constants; that is, the impedance modulus showed an increasing–decreasing–increasing trend with increasing salt spray corrosion time. The peaks of the phase angle of the high and intermediate frequency shift to the low-frequency direction. The Nyquist diagram of 6061Al alloy in Figure 9d shows that in the early stage of salt spray corrosion, the capacitive arc resistance in the high-frequency direction increases slowly with increasing corrosion time. This is because the oxygen in the solution promotes the corrosion products on the surface of the material. However, the rate of film formation is slow in the early stage. The impedance spectrum in the middle and late stages of salt spray corrosion shows a single capacitive impedance arc, indicating that a complete and dense corrosion product film is gradually formed, and the reactants need to pass through the film layer to reach the material matrix to react. The Bode diagram has two time constants in Figure 9e,f. With an increase in the salt spray corrosion time, the impedance modulus value shows a trend of first decreasing by 144 h, increasing by 148 h, then decreasing by 240 h.

### 3.7. Analysis of (Corrosion Rate) CR

CR was calculated by weight loss according to ASTM-G31-72 [28,29,30]:

CR = (K × W)/(A × T × D)(1)

where the constant K = 8.76 × 10^3^, W is the weight loss, A is the sample area exposed to NaCl solution, T is the exposure time, and D is the standard density of tested materials. In this experiment, parallelepiped samples with dimensions of 8 × 8 × 5 mm^3^. The exposed surface of the sample was 64 mm^2^. The density of the SiC_3D_/6061Al was 2.87 g/cm^3^, while density of the 6061Al alloy was 2.73 g/cm^3^.

**Figure 10:**
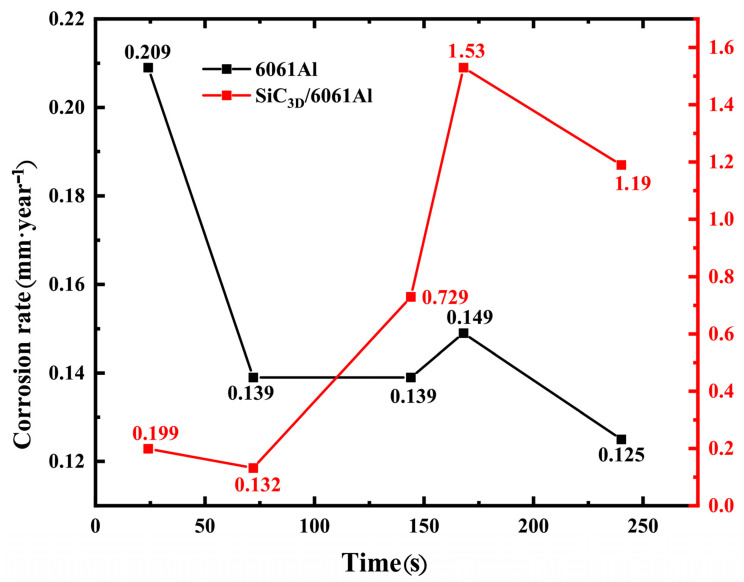
Corrosion rate (CR) of the two materials vs. NSS time.

The CR of two materials vs. NSS time in Figure 10 shows the CR according to weight loss after NSS tests of 6061Al is lower than that of SiC_3D_/6061Al and the weight loss is less than that in SiC_3D_/6061Al, which is consistent with the corrosion morphology and the law of weight loss per unit area. The CR decreased from 0.209 to 0.134 mm/year for 6061Al alloy. However, a constant increase was observed during the 24 and 48 h, with a maximum value of 1.53 mm/year at 168 h of NSS tests. After this time, we recorded a decrease in the CR, at 240 h of NSS tests, reaching values of 1.19 mm/year for SiC_3D_/6061Al. The result indicates that the addition of a skeleton structure of SiC_3D_ results in an increase of CR of 6061Al alloy.

### 3.8. Morphology Analysis of Salt Leaching (SL) Corrosion

**Figure 11:**
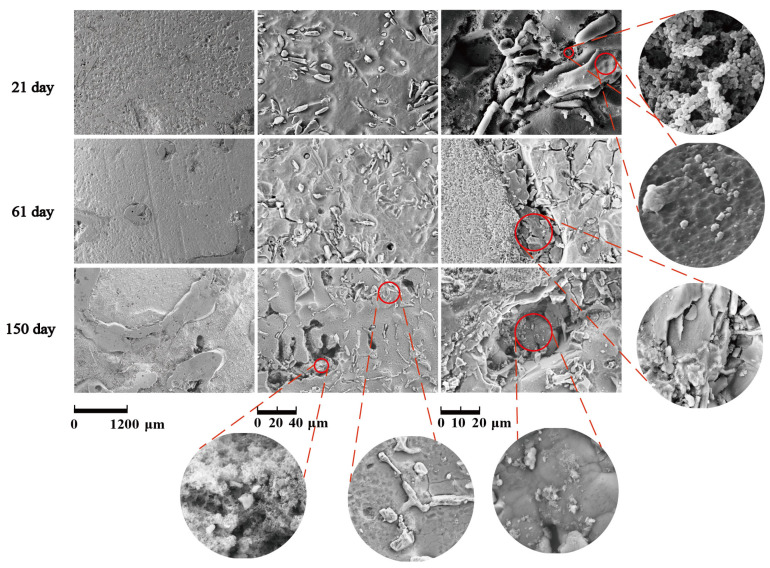
Corrosion morphology of SiC_3D_/6061Al for different salt leaching (SL) times.

**Figure 12:**
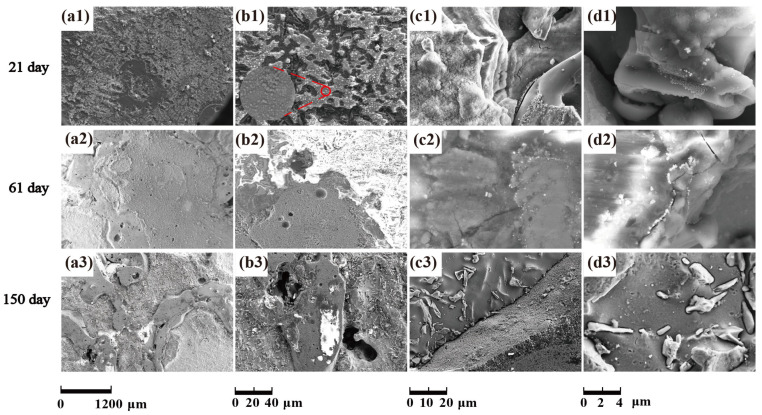
Morphology of corrosion products of SiC_3D_/6061Al for different SL times. (**a1**–**a3**) Corrosion morphology of SiC_3D_/6061Al with low magnification; (**b1**–**b3**) Corrosion morphology of the interface of SiC_3D_/6061Al with low magnification; (**c1**–**c3**) Corrosion morphology of the interface of SiC_3D_/6061Al with high magnification; (**d1**–**d3**) Corrosion morphology of the interface of SiC_3D_/6061Al with the highest magnification.

**Figure 13:**
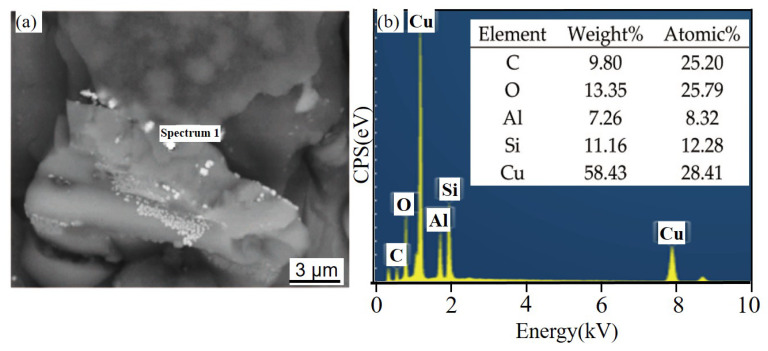
EDS of SiC phase at the interface of SiC_3D_/6061Al after SL for 21 days. (**a**) SEM image of SiC phase; (**b**) EDS of SiC phase.

**Figure 14:**
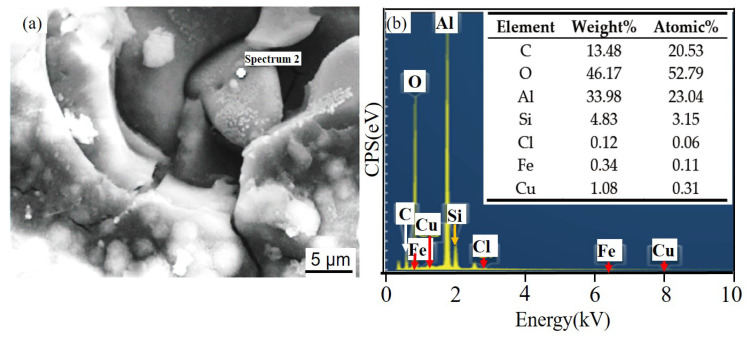
EDS of particle in the corrosion pits of SiC_3D_/6061Al after SL for 21 days. (**a**) SEM image of particles in the corrosion pit; (**b**) EDS of particle.

**Figure 15:**
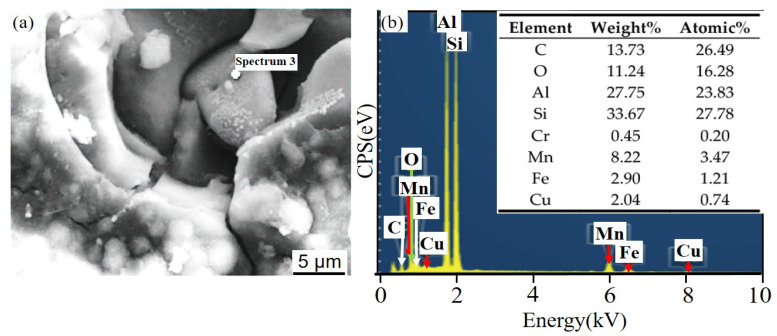
EDS of particles in the corrosion pit of SiC_3D_/6061Al after SL for 21 days. (**a**) SEM image of particles in the corrosion pit; (**b**) EDS of particle.

**Figure 16:**
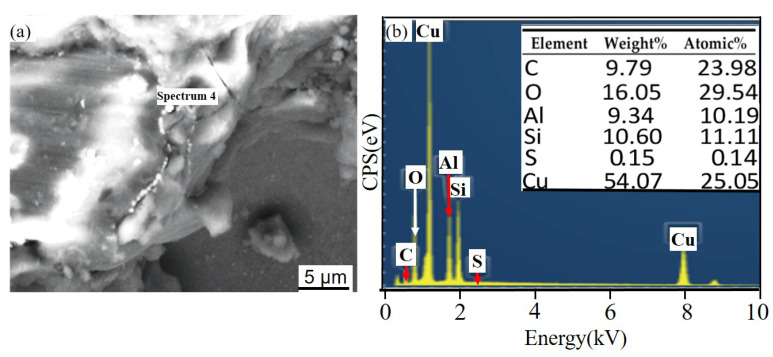
EDS of matrix cracks in SiC_3D_/6061Al after SL for 61 days. (**a**) SEM image of matrix cracks; (**b**) EDS of matrix cracks.

**Figure 17:**
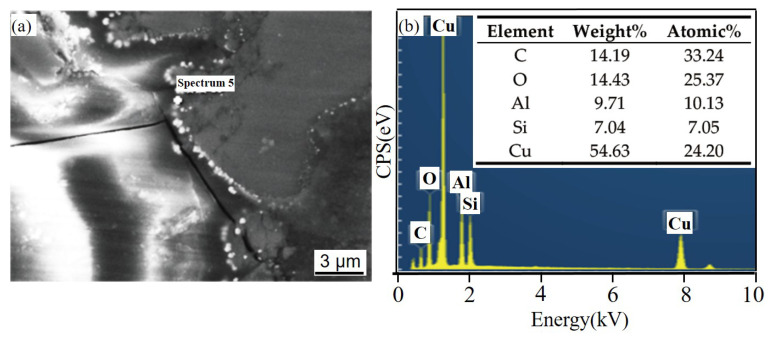
EDS of particles surrounding a circle of SiC_3D_/6061Al after SL for 61 days. (**a**) SEM image of particles surrounding a circle; (**b**) EDS of particles surrounding a circle.

**Figure 18:**
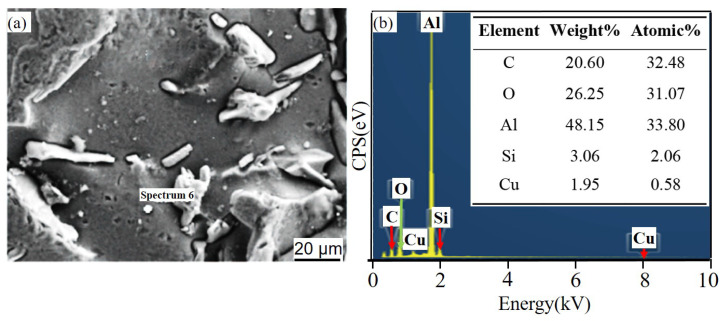
EDS of the interface of SiC_3D_/6061Al after SL for 150 days. (**a**) SEM image of interface; (**b**) EDS of interface.

Figure 11 and Figure 12 show the corrosion morphology and corrosion products morphology of SiC_3D_/6061Al for different SL times. After 21 days of SL, the skeleton structure of SiC_3D_ has been corroded shown in Figure 11. The corrosion products are densely distributed on the surface and interface of the Al metal phase of the SiC_3D_/6061Al shown in Figure 12. Figure 13, Figure 14, Figure 15, Figure 16, Figure 17 and Figure 18 show the EDS of corrosion products at different locations of SiC_3D_/6061Al. From Figure 13, Figure 14 and Figure 15, after 21 days of SL, many particles are observed on the surface of the SiC_3D_/6061Al. EDS analysis show that large particles are Fe-rich phases, and small particles are Cu-rich phases in Figure 14. EDS of smaller particles in the corrosion pit of SiC_3D_/6061Al after SL for 21 days in Figure 15 show smaller particles are mixture of Cu, Fe, Cr and Mn precipitated phases. As the corrosion time increases, an oxide film is formed on the surface of Al metal phase of the SiC_3D_/6061Al after 61 days of Sl. According to Figure 16 and Figure 17, SiC was surrounded by Cu atoms to prevent further expansion of corrosion. The presence of Cu makes the corrosion potential of Al alloy move in the positive direction. When the corrosion time reaches 61 days, there are obvious cracks at the interface in Figure 17. It proves Cu atoms prevent the substrate matrix from being corroded. In 150 days, the oxide film was destroyed, and the Al metal phase was severely corroded in Figure 18.

With the increase of salt immersion time, corrosion pits on the surface of SiC_3D_/6061Al increase and gather, and eventually develop into exfoliation corrosion, which is consistent with the corrosion history of NSS in Figure 12(a1–a3). Corrosion morphology of SiC_3D_/6061Al with low magnification in Figure 12(b1–b3), higher magnification in Figure 12(c1–c3), and with the highest magnification in Figure 12(d1–d3) show the details of the corrosion product morphology of SiC_3D_/6061Al under different salt immersion times. Figure 12(b1–b3) show the Al_2_O_3_ closely covers the surface of the metal phase. Figure 12(c1–c3) show Cu atoms surround the SiC ceramic phase, and the content of Cu, Fe, and Mn at the interface increases. After 150 days of corrosion, the interface has been corroded obviously.

**Figure 19:**
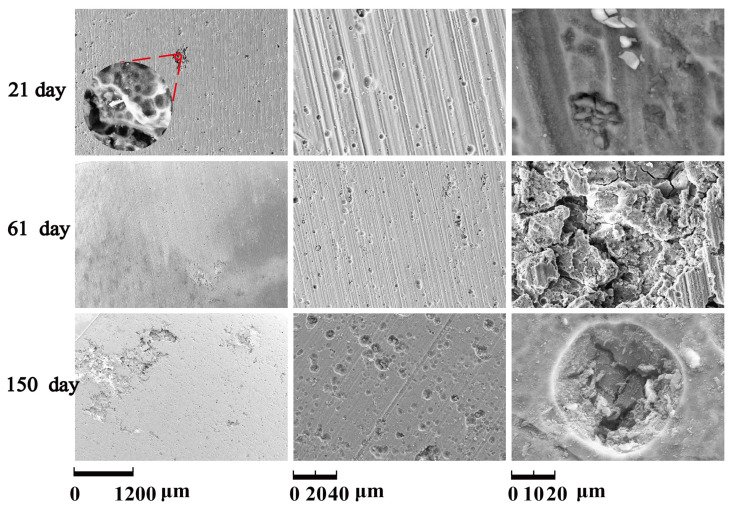
Corrosion morphology of 6061Al alloy.

Figure 19 show the corrosion morphology of 6061Al alloy under different salt immersion times.

**Figure 20:**
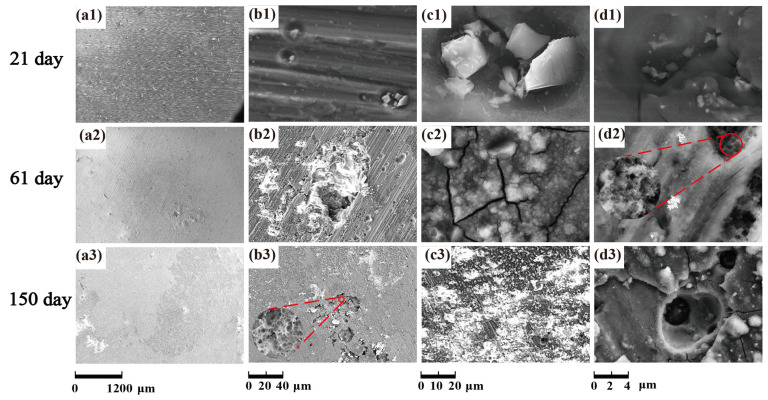
Corrosion product morphology of 6061Al alloy. (**a1**–**a3**) Corrosion morphology of 6061Al with low magnification; (**b1**–**b3**) Corrosion morphology of the interface of 6061Al with low magnification; (**c1**–**c3**) Corrosion morphology of 6061Al with higher magnification; (**d1**–**d3**) Corrosion morphology of 6061Al with the highest magnification.

With the increase of salt immersion time, corrosion pits on the surface of 6061Al alloy increase and gather, and eventually develop into exfoliation corrosion, which is consistent with the corrosion history of NSS in Figure 20(a1–a3). Corrosion morphology of 6061Al with low magnification in Figure 20(b1–b3), higher magnification in Figure 20(c1–c3), and with the highest magnification in Figure 20(d1–d3) show the details of the corrosion product morphology of 6061Al alloy under different salt immersion times.

**Figure 21:**
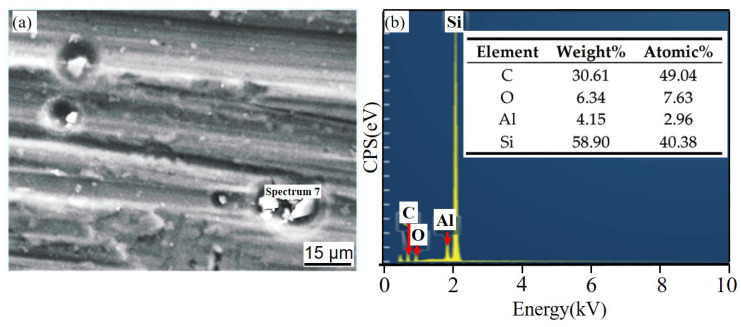
EDS of corrosion products in corrosion pits of 6061Al alloy after SL for 21 days. (**a**) SEM image of corrosion products; (**b**) EDS of corrosion products.

Figure 21 shows the EDS of corrosion products on the surface of 6061Al alloy by SL for 21 days. After analysis, the corrosion products are mainly Al_2_O_3_, AlCl_3_, and Mg_2_Si.

**Figure 22:**
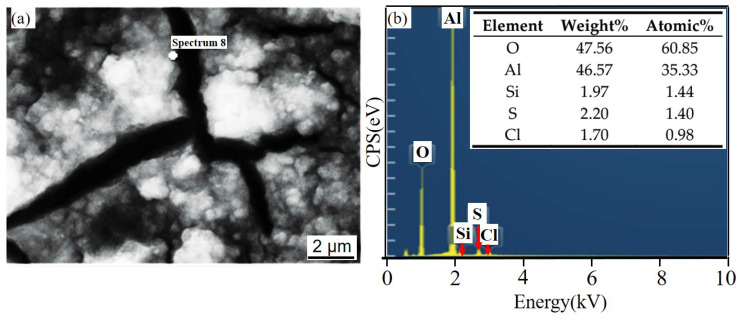
EDS of matrix cracks in 6061Al alloy after SL for 61 days. (**a**) SEM image of matrix cracks; (**b**) EDS of matrix cracks.

Figure 22 shows the corrosion products of 6061Al alloy pitting pits after SL for 61 days. It can be seen that Cr is the main cause of cracks in the material, and the EDS corrosion products are mainly Al_2_O_3_, SiC, Cu, and AlCl_3_.

**Figure 23:**
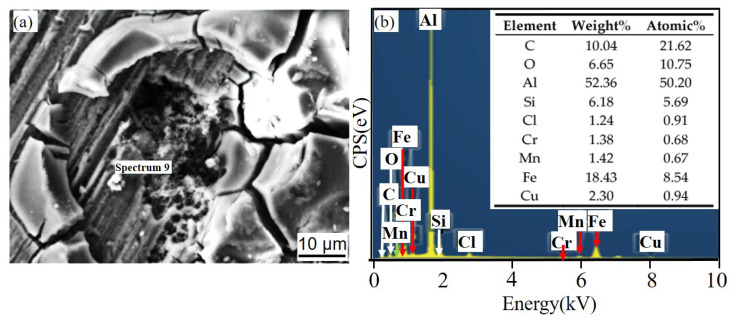
EDS of particle in 6061Al alloy corrosion pits after SL for 61 days. (**a**) SEM image of particle; (**b**) EDS of particle.

Figure 23 shows the large particle aggregates at d_3_ and the EDS of small particles. The analysis shows that the large particles are Fe-rich, and the small ones are Cu-rich, which are consistent with SiC_3D_/6061Al.

**Figure 24:**
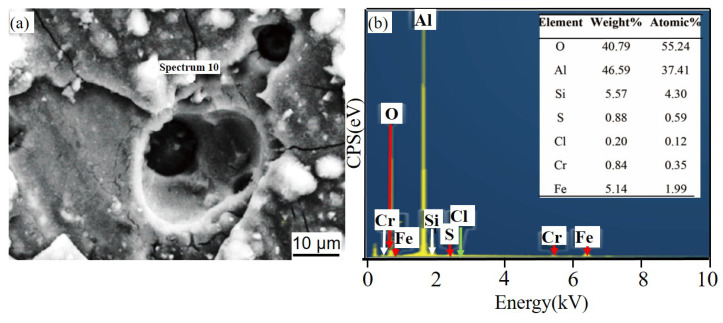
EDS of matrix cracks in 6061Al alloy after SL for 150 days. (**a**) SEM image of matrix cracks; (**b**) EDS of matrix cracks.

**Figure 25:**
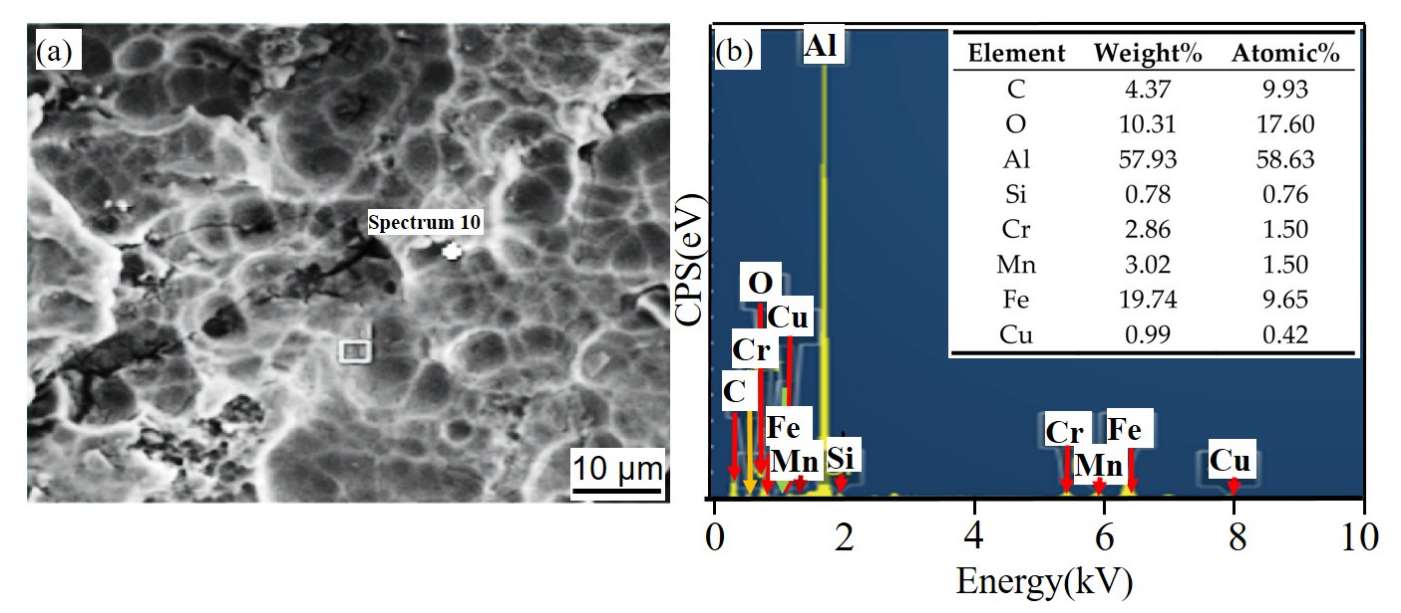
EDS of 6061Al alloy in corrosion pit after SL for 150 days. (**a**) SEM image of corrosion pit; (**b**) EDS of corrosion pit.

Figure 24 and Figure 25 show that the content of Cr and Fe in the spalling pits is greater than that in the holes, and the dark-colored 6061Al alloy further develops spalling corrosion.

Continuous precipitated phases are observed at the grain boundary of 6061Al alloy. These precipitated phases are Fe-rich and Cr-rich phases, which increase the potential in a small local area, generate a potential difference with the surrounding matrix, and promote the occurrence of galvanic corrosion. The interfaces between second phase particles and 6061Al alloy are kinds of defects. Therefore, on the sites where the second phase particles are detached, localized types of corrosion such as pits can occur. Two main reactions occur at the anode site:

Al ⇌ Al^3+^ + 3 e^−^ (2)

Al^3+^ + 3H_2_O ⇌ Al(OH)_3_ + 3H^+^ (3)

Equation (4) shows that a more acidic (pH = 3–4) environment is created at the anode site. Cl^−^ promotes the anode dissolution of aluminum to form aluminum chloride. The latter is hydrolyzed to form Al(OH)_3_ and acids, thereby converting the pH value to an acid value. Equations (5)–(7) show the possible reactions at the cathode site:

AlCl_3_ + 3H_2_ ⇌ Al(OH)_3_ + 3HCl (4)

3H^+^ + 3e^−^ ⇌ ^3^/_2_H_2_ (5)

^1^/_2_O_2_ + H_2_O + 2e^−^ ⇌ 2OH^−^ (6)

As shown in Equation (6), the cathode position is more alkaline due to the formation of local hydroxides. The presence of oxygen is essential for pitting corrosion. As shown in Figure 20(c2,d3), the aluminum hydroxide precipitates outside the pit, and a cone-shaped corrosion product deposit is formed at the mouth of the pit.

## 4. Discussion

The OCP, PDP curves, and EIS show that SiC_3D_/6061Al corrosion resistance performance is better than that of 6061Al alloy, but the conclusions drawn from the NSS tests are the opposite. We explain the reasons for the different experimental results and analyze the corrosion mechanism of the SiC_3D_/6061Al and 6061Al alloy with different tests.

The destruction of materials by corrosion is affected by two determinants: the CR related to corrosion kinetics and the corrosion trend. For long-term immersed materials, the influence of corrosion products on the CR is very important. The corrosion products on the surface of the material have a protective effect on the substrate, but with extended corrosion time, the corrosion products’ protective effect on the matrix is weakened. Compared with 6061Al alloy, the lower CR of SiC_3D_/6061Al can be partly attributed to the formation of a corrosion-resistant and oxidation-resistant network by the SiC_3D_ skeletons. SiC_3D_ skeletons remain inert in the corrosive solution, which will not promote local electrochemical effects.

Moreover, SiC_3D_ skeletons will reduce the surface area of the metal exposed in the solution, decreasing the CR.

However, SiC_3D_/6061Al produces porosity and shrinkage cavities at the interface during the casting process, and then pitting corrosion is more likely to occur at the interface. The 6061Al alloy generates an oxide film due to the secondary reaction, slowing the corrosion tendency.

Corrosion tendency is expressed by corrosion potential, and a higher corrosion potential indicates a lower corrosion tendency. Electrochemical test results reveal that the corrosion resistance of the SiC_3D_/6061Al composite is higher than that of the 6061Al alloy, indicating that SiC_3D_ will reduce the driving force of corrosion. Therefore, the corrosion tendency of the SiC_3D_/6061Al interface is higher than that of the 6061Al alloy, while the corrosion tendency of the metal phase in SiC_3D/_6061Al is the lowest.

## 5. Conclusions

1. The OCP, PDP curves, and EIS tests on the uncorroded samples show that the corrosion resistance of SiC_3D_/6061Al is better than that of 6061Al.
2. NSS and SL tests show the corrosion degree of SiC_3D_/6061Al and 6061Al is similar in the early corrosion stage, and the corrosion resistance of the metal phase of SiC_3D_/6061Al is higher than that of 6061Al in the later corrosion stage. However, evident corrosion at the interface leads to an increase in the overall CR of the SiC_3D_/6061Al.
3. The EIS curves of SiC_3D_/6061Al and 6061Al after NSS corrosion show the capacitive arc radius of the two materials increased, decreased, and finally increased with increasing corrosion time.
4. The corrosion resistance performance of SiC_3D_/6061Al is better in electrochemical tests; however, the NSS and SL tests show that the corrosion resistance performance of 6061Al alloy is better, and the addition of a skeleton structure of SiC_3D_ results in an increase of CR of 6061Al alloy in the NSS tests.
5. The second phase determines the CR of the 6061Al alloy, and the CR of the SiC_3D_/6061Al composite material is mainly determined by the corrosion products.

## Figures and Tables

**Table 1:** Composition of 6061Al alloy (mass fraction).

Elements	Mg	Si	Cu	Mn	Fe	Zn	Ti	Cr	Al
Wt%	0.95	0.75	0.20	0.15	0.55	0.15	0.05	0.08	Balance

**Table 2:** Corrosion potential and corrosion current.

Sample	Open Circuit Potential OPC (mV)	*E*_corr_ (mV)	*I*_corr_ (μA·cm^2^)
SiC_3D_/6061Al	−651.9	−663.5	26.04
6061Al	−695.6	−691.6	60.13

## Data Availability

Data sharing is not applicable for this article.
